# Survival After Transarterial Radioembolization in Patients with Unresectable Intrahepatic Cholangiocarcinoma: An Updated Meta-analysis and Meta-regression

**DOI:** 10.1007/s00270-024-03825-7

**Published:** 2024-08-26

**Authors:** Maria Adriana Cocozza, Elton Dajti, Lorenzo Braccischi, Francesco Modestino, Peter Reimer, Alessandro Cucchetti, Giovanni Barbara, Cristina Mosconi

**Affiliations:** 1 Division of Interventional Radiology, IRCCS Azienda Ospedaliero-Universitaria di Bologna, Bologna, Italy; 2 Gastroenterology Unit, IRCCS Azienda Ospedaliero-Universitaria di Bologna, Bologna, Italy; 3 Department of Medical and Surgical Sciences (DIMEC), University of Bologna, Bologna, Italy; 4 Department of General and Oncologic Surgery, Morgagni-Pierantoni Hospital, AUSL Romagna, Forlì, Italy; 5 Städtisches Klinikum Karlsruhe, Institute for Diagnostic and Interventional Radiology, Academic Teaching Hospital the University of Freiburg, Moltkestraße 90, 76133 Karlsruhe, Germany

**Keywords:** Radioembolization, Cholangiocarcinoma, Selective internal radiation therapy, Meta-analysis, Meta-regression

## Abstract

**Purpose:**

Transarterial radioembolization (TARE) has emerged as a promising therapeutic approach for unresectable intrahepatic cholangiocarcinoma (ICCA). We updated our previous meta-analysis with meta-regression to explore the efficacy of TARE in the context of ICCA.

**Methods:**

We searched PubMed and Scopus for studies published up to September 1, 2023. The primary outcome was overall survival. Secondary outcomes were tumor overall response rate, severe adverse events, and downstaging to surgery. Meta-analysis employed a random-effects model, and meta-regression was utilized to explore sources of heterogeneity.

**Results:**

We included 27 studies, involving 1365 patients. Pooled survival estimates at 1, 2, and 3 years were 52.6%, 27%, and 16.8%, respectively. Meta-regression revealed that the proportion of patients naïve to treatment was the only pre-TARE predictor of survival (1-, 2-, and 3-year survival of 70%, 45%, and 36% for treatment-naïve patients, mean survival 19.7 months vs. 44%, 18%, and 7% for non-naïve patients, mean survival 12.2 months). Overall response according to RECIST 1.1 and mRECIST was 19.6% and 67%, respectively. Effective downstaging to surgery was possible in varying rates (3–54%); the mean survival in these patients was 34.8 months (1-, 2-, and 3-year survival of 100%, 87%, and 64%). About 45.7% of patients experienced adverse events, but only 5.9% were severe.

**Conclusions:**

Our study benchmarked the survival rates of patients undergoing TARE for unresectable ICCA and showed that this is a valid option in these patients, especially if naïve to previous treatments. Downstaging to surgery is feasible in selected patients with promising results.

**Graphical Abstract:**

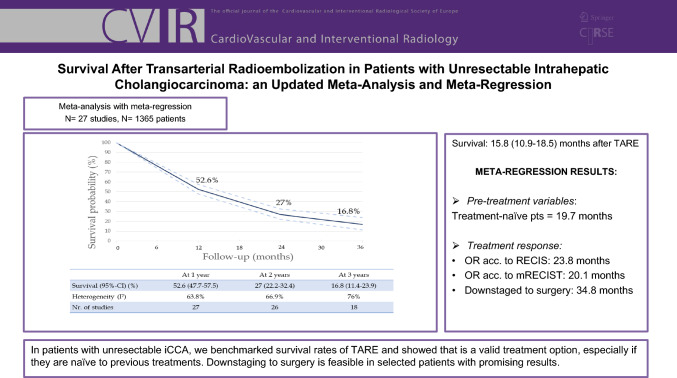

**Supplementary Information:**

The online version contains supplementary material available at 10.1007/s00270-024-03825-7.

## Introduction

Intrahepatic cholangiocarcinoma (ICCA) is a rare and aggressive type of liver cancer that arises from the bile ducts within the liver. ICCA ranks as the second most prevalent primary liver cancer, following hepatocellular carcinoma, accounting for less than 10% of cholangiocarcinomas [1, 2], but its incidence is rising. At present, hepatic resection represents the only potentially curative option, presenting a 10% chance of survival and disease-free status a decade post-treatment [3]. However, only 30–40% of ICCAs are diagnosed early enough to qualify for a curative resection. In unresectable ICCAs, the prognosis is poor, but several treatment options are available [4].

Chemotherapy is often the first line of treatment for inoperable ICCA. The combination of gemcitabine and cisplatin has been shown to offer some benefit in terms of tumor shrinkage and symptom relief, but the survival benefit and response rates are limited. The median progression-free survival with this regimen is merely 8 months, with an overall median survival of less than a year in more advanced cases [5, 6].

“TOPAZ-1” is the first phase 3 trial to demonstrate the benefit of immunotherapy, in particular durvalumab, in patients with biliary tract cancer reporting 24-month overall survival rate of 24.9% [7]. The introduction of immunotherapy represents a revolutionary treatment modality which might change the landscape of ICCA management, but more data from clinical trials are eagerly awaited [8–11].

Such dire survival statistics have driven specialists toward exploring multimodal and combined therapeutic approaches. Among these, intra-arterial therapies (IATs), such as transarterial chemoembolization (TACE) and transarterial radioembolization (TARE), have emerged as promising strategies.

Our previous meta-analysis [12], which included only nine studies, revealed encouraging results for patients with unresectable ICCA undergoing TARE, showing 1-, 2-, and 3-year pooled survival rates of 55.7%, 33.1%, and 20.2%, respectively. While these results were promising, recent studies have emerged over the past few years that further support and strengthen these findings. Notably, the phase 2 clinical trial conducted by Edeline et al. [13] emphasized the benefit of a combined approach involving first-line chemotherapy alongside TARE. This combination enabled downstaging to surgery in a significant proportion (22%) of patients, and the median overall survival was 22 months. These results support the inclusion of TARE in the treatment flowchart for patients with ICCA. However, the role of locoregional therapies in the guidelines remains unclear, as evidenced by the 2023 guidelines from the European Association for the Study of the Liver (EASL) [14], and the recommendation to support their use is weak.

We aimed to update our previous meta-analysis with meta-regression [12] and provide new benchmarks for the survival rates after TARE in patients with unresectable ICCA.

Secondary aims were to (i) evaluate the impact of patients’ and treatment’s characteristics on survival through meta-regression analysis and (ii) assess rates of tumor response and successful downstaging leading to surgical intervention and their impact on the primary outcome.

## Methods

### Literature Search Strategy

A systematic exploration of articles on radioembolization for ICCA, published until July 31, 2023, has been conducted using PubMed and Scopus databases. There were no restrictions on the starting date of the articles included in the search. The meta-analysis adhered to both the guidelines outlined in the Meta-analysis of Observational Studies in Epidemiology and the PRISMA guidelines. For further information and specifics, refer to the Supplementary Material 1.

### Literature Screening and Inclusion Criteria

One author (MA.C) initially conducted a screening process to eliminate articles deemed irrelevant based on title, abstract, and publication keywords. The selection of studies proceeded through three levels of screening, as outlined in the Supporting Information. The final inclusion criteria of studies were: (i) a study population comprising patients treated for ICCA with TARE; (ii) a detailed description of the study population included in the studies; and (iii) availability of patient survival rate descriptions for at least 1-year post-TARE. In cases where a subsequent study provided a more comprehensive dataset or included the original dataset, the most recent and comprehensive report was chosen. These linked studies were identified based on authorship, institutions, design, length of follow-up, and study populations. If additional data or results were required, the corresponding author of each report was contacted via email. Any discrepancies in inclusion were resolved through discussions between the reviewers and a third investigator (C.M.).

### Data Extraction and Quality Assessment

We extracted the following data according to a pre-specified sheet: study period and location, study design, study size, patients’ characteristics (age, gender, and performance status), tumor characteristics (burden, extension, multifocality, extrahepatic dissemination, and infiltrative pattern), treatment characteristics (previous treatments, concomitant chemotherapy, and type of microspheres), and clinical outcomes (adverse events, tumor response, downstaging to surgery, and overall survival). Tumor response rate was evaluated according to the response evaluation criteria in solid tumors (RECIST 1.1) criteria [15] and modified mRECIST criteria [16]; overall response rate was defined as complete + partial response rate; downstaging to surgery, refers to tumor shrinkage to satisfy the surgical criteria for resectability. Chemotherapy data were collected when retrieved studies clearly described that it was administered in addition to/after TARE. The quality of each selected study was assessed by two investigators (MA.C. and E.D.) through the Cochrane tool (RoB-2) [17] for randomized controlled trial (RCTs) and the Newcastle–Ottawa scale (NOS) for observational studies [18]. Any divergences were resolved by discussion between reviewers and a third investigator (C.M.).

### Statistical Analysis

The primary outcome measure for the meta-analysis was overall survival after the first TARE procedure. Secondary outcomes measures were considered: (i) tumor overall response rate according to RECIST 1.1 and mRECIST criteria and (ii) the rate of patient undergoing surgery after successful downstaging of the disease.

Demographical characteristics and available clinical and tumor features were pooled together to obtain a description of the joint study population. Dichotomous variables, including survival rates, were estimated as pooled binomial proportions with 95% of confidence interval (C.I.) applying the Freeman-Tukey double arcsine transformation to retain studies with proportions at 0 or 1 margins and ensuring admissible confidence intervals for the pooled proportions. Continuous variables were pooled in weighted means with 95% C.I.; when studies reported this variable as median and range, the mean and variance were estimated as proposed by Wan et al. [19]. Studies were not weighted for their quality. The primary survival endpoints were fixed at 1, 2, and 3 years from TARE. Since most reports did not provide the number of patients at risk or tick-marks on Kaplan–Meier curves for censoring events, we were forced to assume it as a binomial proportion from survival rates as proposed by Tierney et al. [20]. Moreover, we pooled in summary mean survival from TARE both in the overall population and predicted its value through the meta-regression analysis. Statistical heterogeneity was explored by inconsistency (*I*^2^) statistics; the heterogeneity was considered substantial if *I*^2^ > 50% [21, 22]. Since the present meta-analysis was based on studies not identical in their methods and/or the characteristics of the included patients, a meta-regression analysis that included available covariates was performed. Covariates to be tested were selected on the basis of their clinical likelihood to modify the primary outcome measures and their presence in the selected literature. All comparisons were made by the random-effects model of DerSimonian and Laird [23], if not specified otherwise. Two-sided *p* < 0.050 were considered statistically significant. Meta-analysis and meta-regression were performed using the packages “meta” and “metafor” for R-Project 4.1.1.

## Results

### Results of the Literature Search

**Fig. 1:**
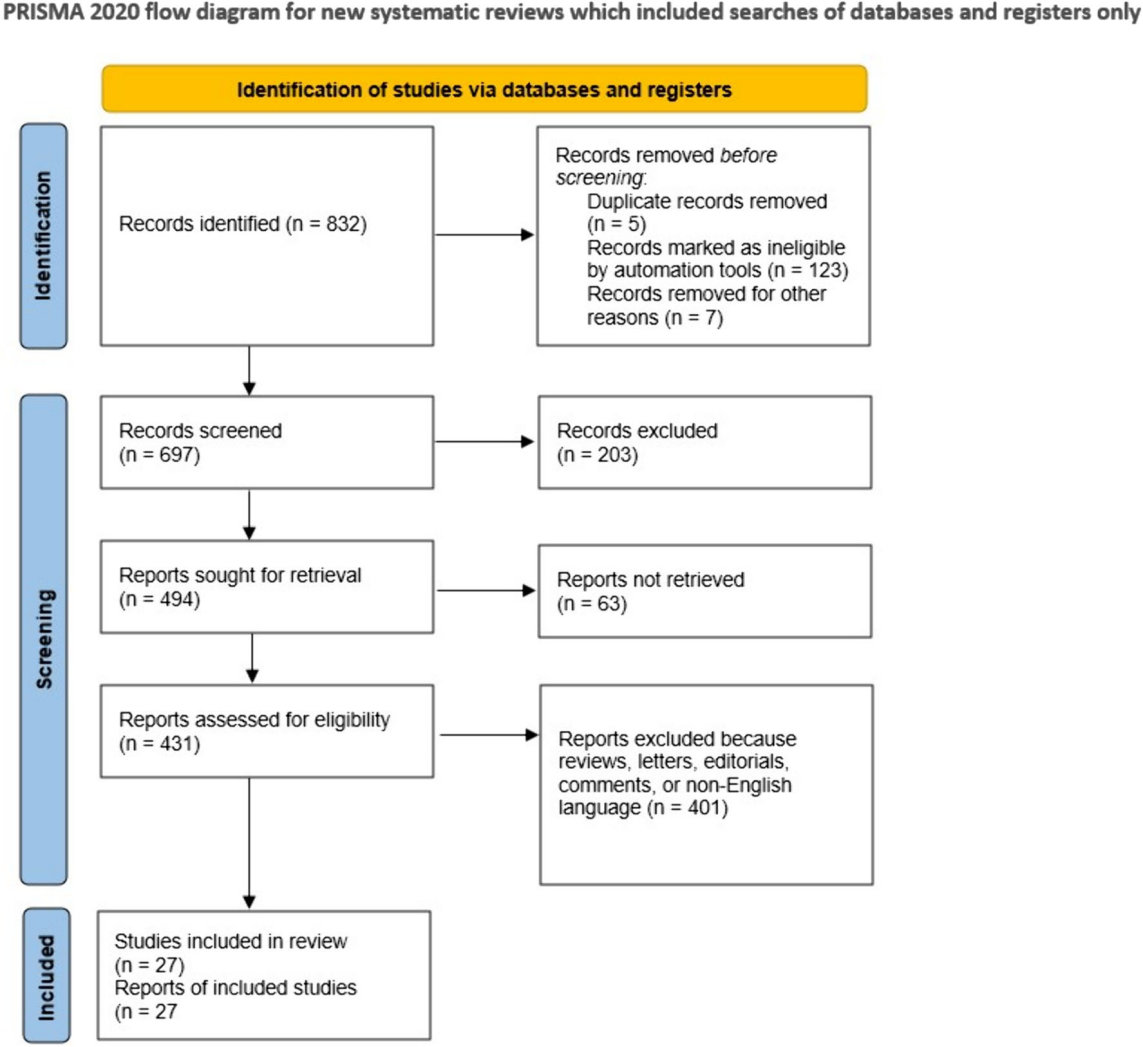
Literature search used in the present analysis outlining the included and excluded studies

A total of 832 articles were initially identified based on our search criteria for screening (Fig. 1). After applying the exclusion criteria, 32 studies were selected following a thorough assessment of the full manuscripts. Five studies were excluded for overlapping cohorts. Consequently, the final list of included studies comprised 27 reports and 1365 patients (Table 1) [13, 24–49]. The quality of the included studied was deemed to be sufficient.

**Table 1:** Summary of studies included in the meta-analysis

Author (year)	Study design	Nr	Type of microspheres	Enrollment period		Survival (months)	NOS/RoB-2
Saxena et al. (2010) [24]	Single center, prospective	25	Resin	Jan 2004	May 2009	20	7
Hoffmann et al. (2012) [25]	Single center, retrospective	33	Resin	Apr 2007	Jan 2010	20	6
Rafi et al. (2013) [26]	Single center, prospective	19	Resin	Dec 2002	Oct 2010	12	8
Camacho et al. (2014) [27]	Single center, prospective	21	Resin	Jan 2009	Dec 2012	16	7
Filippi et al. (2015) [28]	Single center, prospective	17	Resin	N/A	N/A	16	7
Soydal et al. (2016) [29]	Single center, retrospective	16	Resin	Jan 2008	Dec 2014	10	7
Shaker et al. (2018) [30]	Single center, retrospective	17	Resin, glass	Jan 2006	Dec 2016	34	6
Bourien et al. (2018) [32]	Single center, retrospective	64	Glass	Aug 2010	Oct 2016	16	6
Reimer et al. (2018) [31]	Single center, retrospective	21	Resin	Jan 2005	Nov 2016	N/A	8
Gangi et al. (2018) [33]	Single center, retrospective	85	Glass	May 2009	May 2016	12	8
Levillain et al. (2019) [34]	Multicenter, retrospective	58	Resin	Jan 2004	Sep 2018	10	7
White et al. (2019) [35]	Multicenter, prospective	61	Resin, glass	Dec 2013	Feb 2017	9	8
Edeline et al. (2020) [13]	Multicenter, prospective	41	Glass	Nov 2013	Jun 2016	22	Low
Bargellini et al. (2020) [37]	Multicenter, retrospective	81	Resin	Jul 2008	Oct 2017	14,5	7
Buettner et at. (2020) [38]	Multicenter, retrospective	115	Resin, glass	Jun 2006	Feb 2017	11	6
Koehler et al. (2020)	Multicenter, retrospective	46	Resin	N/A	N/A	9,5	7
Sarwar et al. (2021) [40]	Single center, retrospective	31	Resin	Oct 2015	Sep 2020	22	8
Paprottka et al. (2021) [42]	Single center, retrospective	73	Resin	N/A	N/A	14	7
Cheng et al. (2021) [41]	Single center, retrospective	38	Resin, glass	Jan 2013	Dec 2018	11	6
Paz-Fumagalli et al. (2021) [39]	Single center, retrospective	28	Glass	May 2016	Feb 2020	N/A	7
Robinson et al. (2022) [45]	Multicenter, prospective	94	Resin	Jul 2015	Aug 2020	14	8
Gupta et al. (2022) [46]	Single center, retrospective	136	Glass	Jun 2004	Jan 2020	14	8
Schatka et al. (2022) [44]	Single center, retrospective	39	Resin	Jan 2009	Dec 2016	8	7
Kumar et al. (2022) [43]	Single center, retrospective	16	Glass	May 2009	Oct 2019	7	8
Ahmed et al. (2023) [47]	Single center, retrospective	13	Glass	Dec 2018	May 2021	29	6
Schaarschmidt et al. (2023) [49]	Multicenter, retrospective	128	Resin, glass	May 2007	May 2021	12	8
Mosconi et al. (2023) [48]	Single center, retrospective	49	Resin	Jan 2016	Jun 2021	16	8

NOS; Newcastle–Ottawa scale, RoB; risk of bias

### Characteristics of the Included Studies

Seven studies had a prospective design, while the remaining 20 were retrospective. Eight studies were multicenter, of which three were both multicenter and prospective. Fifteen studies used resin microspheres [24–29, 31, 34, 36, 37, 40, 42, 44, 45, 48], seven used glass microspheres [13, 32, 33, 39, 43, 46, 47], and the remaining five used both [30, 35, 38, 41, 49]. The number of included patients ranged from 13 [47] to 136 [46]. The inclusion criteria varied among studies: four studies [13, 31, 39, 47] included only patients naïve to treatment, six studies included only patients previously treated with (and mostly refractory to) chemotherapy [25–27, 34, 41, 42], and the others included both with a rate of treatment-naïve patients varying from 8% [35] to 77% [40].

### Characteristics of Included Patients

The pooled study cohort comprised 1365 individual patients with unresectable ICCA who underwent TARE. We summarized the results of the meta-analysis regarding demographic, clinical, and tumor characteristics in Table 2. Briefly, summary mean age was 64.2 (95% CI 62.6–65.9) years, and summary proportion of men was 49.2% (95% CI 45.8–52.6%). The tumor was bilobar (47.4%, 95% CI 39.4–55.4%) and multifocal (53.5%, 95% CI 45.5–61.4%). Extrahepatic disease, consisting mostly of lymph node metastases, was presented in a summary proportion of 36.2% (95%-29.6–43.4%). In the subgroup of studies (*n* = 13) distinguishing between mass-forming and infiltrative pattern ICCA, the summary proportion of the latter type was 38.4% (95% CI 16.8–65.8%). Noteworthy, the rate of patients naïve to treatment, intended as surgery or any IAT, was 25% (95% CI 8.2–55.7%) and 48.8% (95% CI 24.9–73.3%) received concomitant chemotherapy.

**Table 2:** Pooled analysis of clinical features over study population submitted to radioembolization

Variable	Number of studies	Weighted analysis (95% CI)	*I*^2^ (%)
*Patient characteristics*			
Age (years)	25	64.2 (62.6–65.9)	84.2
Male (%)	27	49.2 (45.8–52.6)	32.4
Performance status < 2 (%)	22	92.3 (83–96.7)	75.7
Naïve to treatment (%)	27	25 (8.2–55.7)	71.9
*Tumor characteristics*			
Burden > 25%	11	46 (38–54.3)	69.3
Bilobar (%)	23	47.4 (39.4–55.4)	74.8
Multifocal (%)	19	53.5 (45.5–61.4)	79.1
Infiltrative pattern (%)	13	38.4 (16.8–65.8)	75.6
Extrahepatic disease (%)	26	36.2 (29.6–43.4)	71.8
Lymph node metastases (%)	22	23.5 (17.1–31.3)	82.4
Distant metastases (%)	22	3.7 (1.2–10.5)	18.1
*Treatment and follow-up data*			
Use of glass microspheres	27	40.2 (37.7–42.9)*	67.7
Any adverse events	21	45.7 (26.2–66.6)	91.4
Severe (grade ≥ 3) adverse events	21	5.9 (3–11.2)	76.5
Concomitant chemotherapy	22	48.8 (24.9–73.3)	89
Objective response (RECIST 1.1)	20	19.6 (13.6–27.3)	80.3
Objective response (mRECIST)	4	67 (57.2–75.5)*	79
Downstage to surgery	27	4.9 (3.9–6.2)*	48.7

*Fixed-effect analysis

CI; confidence intervals, RECIST 1.1; response evaluation criteria in solid tumors, mRECIST; modified RECIST

### Primary Outcome: Overall Survival

**Fig. 2:**
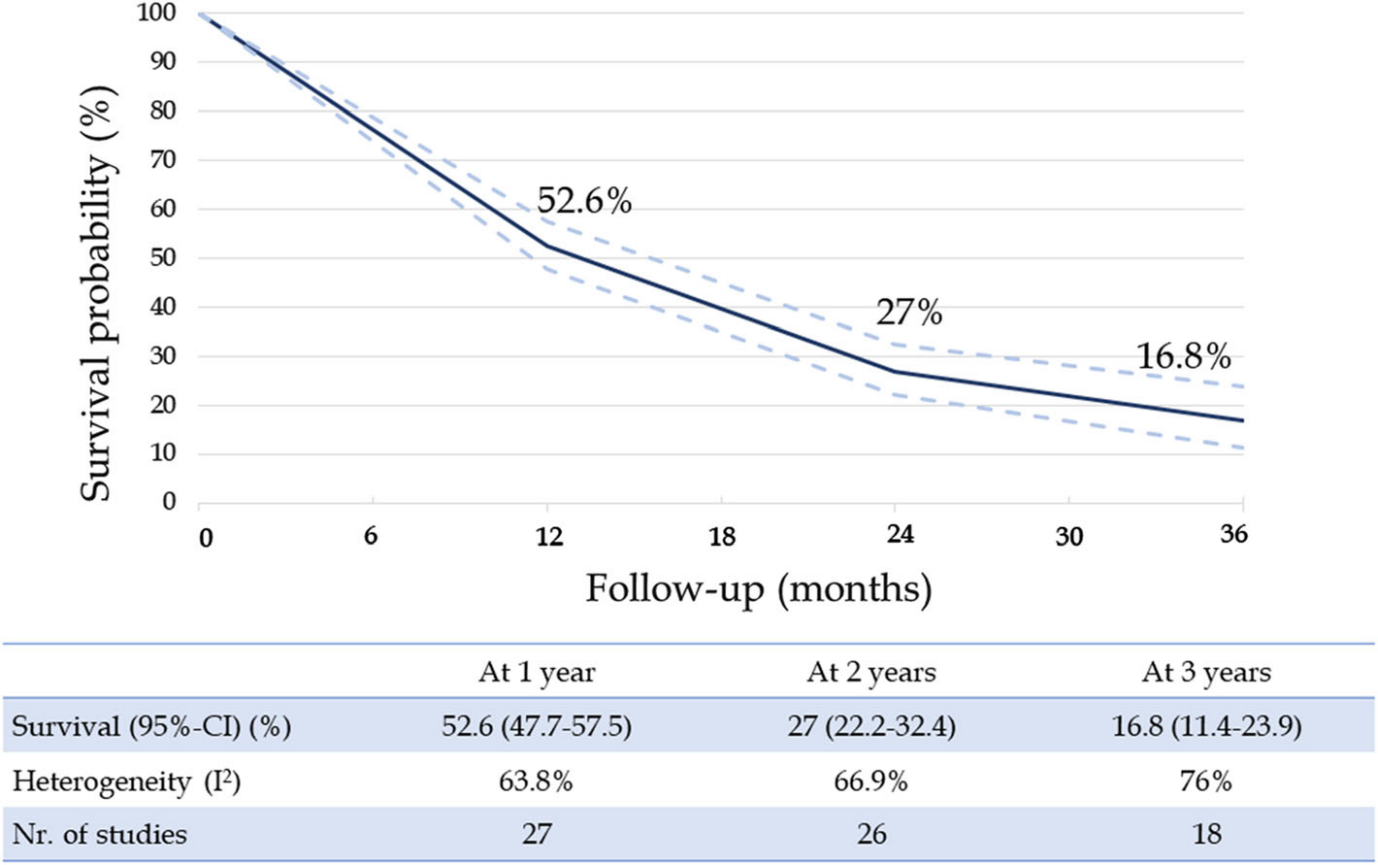
Meta-analysis results for patient survival after TARE

**Fig. 3:**
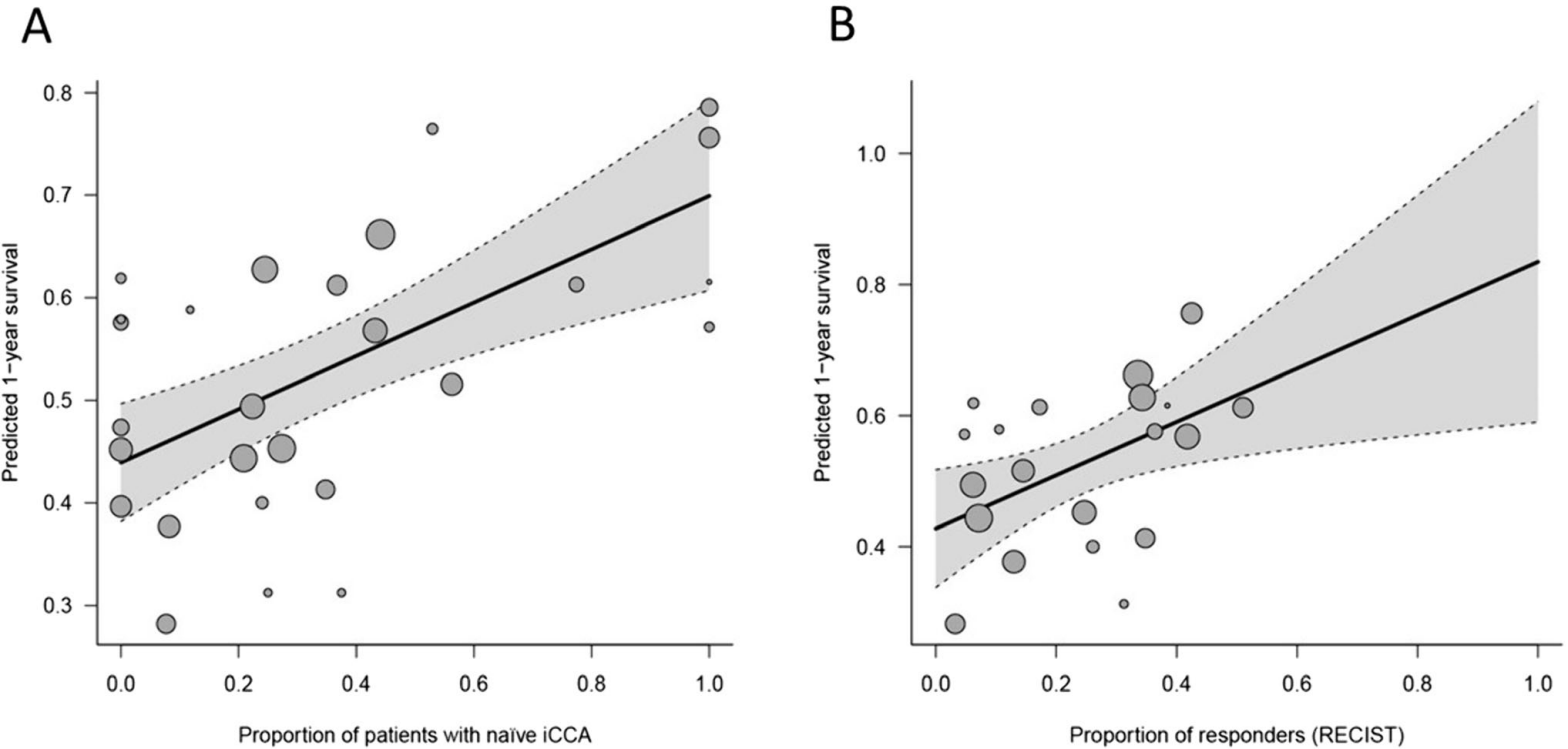
Meta-regression results for patient survival at 1 year after TARE. This figure shows how 1-year survival rates reported in the retrieved literature were influenced by the proportion of patients naïve to treatment (Panel A) and patients achieving overall response according to RECIST 1.1 criteria (Panel B)

The summary survival estimates for 1-, 2-, and 3-year intervals were determined to be 52.6%, 27%, and 16.8%, respectively (Fig. 2), with a mean survival of 15.8 (95% CI 10.9–18.5) months after TARE and of 29 (95% CI 22–33.4) months after diagnosis. However, the heterogeneity between the studies was substantial (> 50%) for all these outcomes. Therefore, we conducted a meta-regression analysis to assess the influence of studies’ and patients’ characteristics on survival. (Table 3). These analyses identified the proportion of patients naïve to treatment as the sole pre-treatment determinant of survival (*p* < 0.001 for all three fixed timepoints) (Fig. 3A); the predicted 1-, 2-, and 3-year survival rates were 70%, 45%, and 36% in treatment-naïve patients and 44%, 18%, and 7% in patients receiving previous treatments. As extreme values at meta-regression, the precited mean survival of treatment-naïve ICCA patients was 19.7 (95% CI 11.5–27.9) months and that of non-naïve patients was 12.2 (95% CI 4.7–19.7) months.

**Table 3:** Results from univariable meta-regression over main outcome considered

Variable	Nr. studies	1-year survival OR (95% CI)	Residual *I*^2^	Nr. studies	2-year survival OR (95%C.I.)	Residual *I*^2^	Nr. studies	3-year survival OR (95% CI)	Residual *I*^2^
*Studies’ characteristics*									
Publication year	27	1.01 (0.95–1.07)	66.4%	26	0.99 (0.91–1.07)	73.0%	18	0.98 (0.86–1.12)	81.0%
Prospective	27	1.12 (0.67–1.86)	66.7%	26	0.77 (0.38–1.54)	73.5%	18	1.24 (0.37–4.16)	81.0%
*Patient characteristics*									
Age (years)	25	1.05 (0.99–1.1)	64.4%	24	1.06 (0.99–1.14)	71.2%	16	1.05 (0.94–1.18)	77.1%
Male (%)	27	3.83 (0.54–26.96)	62.8%	26	6 (0.93–72.39)	70.5%	18	3.57 (0.06–203.57)	81.2%
Performance status < 2 (%)	22	0.55 (0.16–1.55)	60.3%	21	0.57 (0.17–1.96)	58.8%	14	0.98 (0.03–31.12)	66.0%
Naïve to treatment (%)	27	2.79 (1.64–4.77)	43.7%	27	3.27 (1.76–6.07)	53.5%	18	5.85 (2.25–15.**23)**	62.6%
*Tumor characteristics*									
Burden > 25%	11	0.31 (0.04–2.6)	63.6%	10	0.75 (0.04–14.03)	66.2%	7	0.26 (0.01–10.75)	36.8%
Bilobar (%)	23	0.58 (0.16–2.03)	66.2%	22	0.54 (0.12–2.52)	71.3%	15	0.33 (0.03–3.85)	81.4%
Infiltrative pattern (%)	13	0.44 (0.16–1.2)	58.9%	12	0.45 (0.13–1.5)	64.2%	8	0.25 (0.03–2.18)	71.4%
Extrahepatic disease (%)	26	0.5 (0.14–1.76)	65.0%	25	0.62 (0.12–3.33)	74.3%	17	1.14 (0.06–23.48)	82.7%
Lymph node metastases (%)	22	0.47 (0.12–1.94)	68.5	21	1.21 (0.18–8.08)	78.4%	15	0.59 (0.02–19.81)	84.7%
Distant metastases (%)	22	1.27 (0.16–10.21)	70.8%	21	0.54 (0.04–7.6)	78.0%	15	2.06 (0.03–152.78)	84.6%
Treatment data									
Use of glass microspheres	27	1.4 (0.89–2.19)	63.3%	26	1.48 (0.84–2.61)	71.5%	18	2.36 (0.99–5.59)	76.0%
Concomitant chemotherapy	22	1.63 (0.77–3.46)	70.3%	21	2.19 (0.88–5.42)	73.0%	14	2.38 (0.53–10.72)	79.0%

^*^Indicates *p* < 0.05

CI; confidence interval; OR; odds ratio

### Secondary Outcomes: Tumor Response and Downstaging to Surgery

Tumor response was evaluated with imaging (CT and MR) according to RECIST 1.1 and mRECIST in 20 and four studies, respectively. The summary proportion of patients achieving objective response according to RECIST 1.1 criteria rate was 19.6% (95% CI 13.6–27.3%), and it was associated with an improved 1-year survival at meta-regression analysis (OR 5.01, 95% CI 1.43–17.6, p-value = 0.01) (Fig. 3B). In responders, the predicted 1-, 2- and 3-year survival rates were 83%, 51%, and 37% and the predicted mean survival was 23.8 (95% CI 10.6–36.9) months. In non-responders, these rates dropped to 43%, 18%, and 8% and the predicted mean survival was 11.6 (95% CI 2.6–20.6) months. At meta-regression analysis, no pre-treatment variable (type of microspheres and concomitant chemotherapy) was associated with the objective rate (data not shown).

The summary proportion of patients achieving objective response according to mRECIST criteria was 67% (95% CI 57.2–75.5%), and it was also associated with increased 1-year survival (p = 0.03). The precited 1-, 2-, and 3-year survival rates in responders were 81%, 63%, and 57% in responders (mean survival 20.1 months) and 16%, 0%, and 0% in non-responders (mean survival 4.8 months).

Finally, we evaluated the rate of successful downstage to surgery, which was 0% in 16 studies, and it ranged from 3% [41] to 54% [47] in the other studies (summary proportion 4.9%, 95% CI 3.9%-6.2%). At meta-regression analysis, the increasing proportion of patients successfully downstaged to surgery (i.e., hepatic resection) was associated with increased 1-year survival (OR 8.25, 95% CI 1.58–42.91, p = 0.01). At extreme values, the predicted 1-, 2-, and 3-year survival in patients undergoing surgery was estimated 100%, 87%, and 64%, with a predicted mean survival of 34.8 (95% CI 20–49.6) months.

## Discussion

This updated meta-analysis benchmarked the prognosis of patients with ICCA undergoing TARE; the summary overall survival estimates at 1-, 2-, and 3-years after TARE were, respectively, 53%, 27%, and 17%, and the mean survival was 15.8 (95% CI 10.9–18.5) months. These estimates confirm our preliminary findings in a larger sample (27 vs. 9 studies) and more importantly are relatively higher than the survival rates of patients undergoing chemotherapy (10.9 months, 95% CI 9.9–11.6) or immunotherapy (12.7 months, 95% CI 11.5–13.6) according to a recent phase 3 trial on immunotherapy in patients with unresectable biliary tract cancer (60% of enrolled patients had ICCA) [7]. Conversely, the TOPAZ-1 trial [50] included patients with different types of cholangiocarcinoma and at a more advanced stage, and this clearly influences the differences in survival found between systemic therapy and TARE.

We observed that the assessed clinical outcomes exhibited substantial between-studies heterogeneity (> 50%). To elucidate the sources of this heterogeneity, we conducted a comprehensive meta-regression analysis, examining the potential influence of both study and patient characteristics on overall survival. We found that the proportion of patients naïve to treatment emerged as the sole pre-treatment determinant significantly impacting survival (*p* < 0.001 for all three fixed timepoints). Predicted survival rates at 1-, 2-, and 3-year intervals underscored this distinction, with rates of 70%, 45%, and 36% in treatment-naïve patients and with an estimated mean survival as high as 19.7 months (95% CI 11.5–27.9) compared to 44%, 18%, and 7% in those who had received previous treatments (summary mean survival 12.2 (95% CI 4.7–19-7) months). These findings provide critical insights for clinical decision making and rationale of treatment combinations. Of note, in the only phase 2 RCT trial evaluating TARE in patients with ICCA naïve to treatment and receiving concomitant chemotherapy [13], the median overall survival was 22 months (95% CI 14–52), with overall survival rates of 75% at 1 year and 45% at 2 years. These results are strikingly similar to our estimates in treatment-naïve patients, so our study provides real-life evidence supporting the survival benefit of this strategy and its implementation in clinical practice. For this reason, candidate selection of TARE is a crucial aspect. This point involves tumor burden, hepatic function, extrahepatic disease, and overall health. Literature indicates that chemotherapy and the latest systemic treatments alone have demonstrated lower survival rates compared to TARE in treatment-naïve patients [45, 51]. Despite the heterogeneity of patient populations in these studies, which may partially impact outcomes, the higher efficacy of radioembolization in terms of survival in treatment-naïve patients could be attributed to better local tumor control, stimulation of tumor-specific immune responses by releasing tumor antigen, and a higher rate of unresectable lesions being downstaged to surgery [51, 52]. In fact, recent observational studies have focused on the combination of TARE with systemic therapy. Reimer et al. [53] reported that patients who received TARE and concomitant systemic therapy showed better results in overall survival, progression-free survival (PFS), and hepatic PFS compared to treatment-naïve patients or those who received one or more cycles of chemotherapy. These results were similar in the RESiN study [45] for concomitant chemotherapy, while studies on immunotherapy plus TARE are lacking in the literature to our knowledge. Moreover, in our previous meta-analysis [12], we identified treatment-naïve patients with mass-forming ICC as the best candidates for TARE, rather than patients with infiltrative ICC or those who had undergone cycles of chemotherapy.

Regarding the choice of optimal TARE technique, in our meta-analysis, both types of microspheres (resin and glass microsphere) were used in included trials, and no difference was found in terms of prognosis. It is proved that they had different cutoffs of delivered dose for tumor target and liver, but this does not influence survival rates or toxicity [38, 41, 54].

Within the realm of IAT, both TACE and TARE are viable options for treating ICCA within IAT. Conventional TACE (c-TACE) and drug-eluting beads TACE (DEB-TACE) are two modalities, with DEB-TACE possibly offering better tumor response and disease control, though its impact on overall survival is unclear [55]. The choice between TACE and TARE for unresectable ICCA is debated, as the median survival rates are similar. But, TARE was associated with a lower rate of adverse events than TACE. [56, 57]. Regarding the safety profile, TARE is confirmed as a well-tolerated treatment for cholangiocarcinoma, with frequent mild side effects, such as temporary nausea, vomiting, and abdominal pain. Our analysis indicates that adverse events occur in 45.7% of cases, but only 5.9% of these are severe. While severe side effects are rare, the potential for complications like radiation-induced liver and lung disease and non-target gastrointestinal embolization underscores the importance of patient selection, comprehensive pre-procedural planning, and rigorous post-procedural follow-up.

In examining secondary outcomes, our analysis delved into tumor response and downstaging to surgery. These analyses were not feasible in the previous meta-analysis due to the limited number of studies reporting the data and therefore represent a novel finding of our study.

According to RECIST 1.1 criteria, one out five patients had an objective response. We could not identify pre-treatment factors associated with this outcome, but we were able to confirm the prognostic value of such definition and the survival benefit it confers. Responders within this category exhibited a predicted mean survival of 23.8 (95% CI 10.6–36.9) months (vs. 11.6 months, 95% CI 2.6–20.6, in non-responders), and the predicted 1-, 2-, and 3-year survival rates were 83%, 51%, and 37%. On the other hand, two-thirds of the patients achieved an objective response according to mRECIST criteria; responders displayed predicted 1-, 2-, and 3-year survival rates of 81%, 63%, and 57%, with a mean survival of 20.1 months. However, the number of studies reporting this information was limited (*n* = 4), so these data should be interpreted with caution. In light of these compelling results, there arises a pertinent question regarding the prognostic validation of the mRECIST criteria in comparison with RECIST 1.1. The data suggest that both criteria are valuable, but mRECIST might indicate an enhanced prognostic value for treatment outcomes and associated survival benefits. Future validation could potentially establish it as a more reliable tool for predicting patient outcomes.

Radioembolization shows promise as a transformative treatment for ICCA, potentially downstaging tumors to make them resectable and improve survival rates. However, the success of downstaging varies (3–54%), with a large heterogeneity across centers. This estimate might be understated since, in some studies, TARE was offered as a palliative therapy after multiple chemotherapy failures, making downstaging neither an aim nor a possibility. Nevertheless, survival data are very promising: The predicted mean survival was 34.8 months, with 1-, 2-, and 3-year survival rates of 100%, 87%, and 64%, respectively. Despite data heterogeneity and preliminary findings, downstaging to surgery remains a significant predictor of improved survival.

Our study has many limitations: First, there was a high heterogeneity among the baseline clinical and tumor features of the patients included in the retrieved studies. The heterogeneity remained substantial even after the meta-regression analysis. This likely mirrors the inherent diversity within the group of patients subjected to various previous treatments, encompassing surgical interventions, locoregional therapies, and the number of failed chemotherapy lines, among other factors. The profound differences in patient profiles, such as those undergoing radioembolization for post-surgical recurrence versus those opting for TARE due to progression after exhausting all available chemotherapy lines, contribute significantly to this heterogeneity. The heterogeneity complicates the interpretation of long-term outcomes across published experiences, posing challenges for comparing TARE outcomes with standard care and selecting the most appropriate treatment beyond established guidelines. Future research efforts may benefit from further refinement of patient categorization and increased granularity in data collection to address these inherent limitations.

In conclusion, out meta-analysis benchmarked the survival outcomes post-TARE across various clinical contexts. The results suggest that treatment-naïve ICCA patients, especially when assessed with mRECIST criteria, exhibit the most favorable outcomes, indicating promising downstaging effects and providing new possibilities for managing inoperable ICCA. Since the introduction of immunotherapy will revolutionize the management of patients with advanced biliary tract cancer, future studies should investigate the benefit of combining immunotherapy with TARE:

## Supplementary Information

Below is the link to the electronic supplementary material.

Supplementary file1 (DOCX 15 KB)
