# Supplementary material for: Survival After Transarterial Radioembolization in Patients with Unresectable Intrahepatic Cholangiocarcinoma: An Updated Meta-analysis and Meta-regression
Source: Cardiovasc Intervent Radiol. 2024 Aug 26;47(10):1313–24. doi: 10.1007/s00270-024-03825-7 (PMC11486776; doi:10.1007/s00270-024-03825-7)
Supplement: Supplementary file 1 — Supplementary file1 (DOCX 15 KB) [file 270_2024_3825_MOESM1_ESM.docx]

**SUPPLEMENTARY MATERIALS**

**Literature Search Strategy:** The medical subject headings (MeSH) "cholangiocarcinoma," "infusion intra-arterial," and "radiotherapy" were employed, supplemented by the following keywords for further refinement: "radio-embolization" and "selective internal radiotherapy." Results obtained from both electronic databases were systematically compared to generate a unified list of articles for subsequent screening. Additional scrutiny involved a search in the International Clinical Trials Registry Platform (ICTRP), encompassing clinicaltrials.gov, with no additional studies identified. A parallel effort to explore grey literature through Google Scholar similarly yielded no additional available studies.

**Literature Screening:** One author (MA.C) conducted the initial screening to identify articles that were clearly irrelevant based on their title, abstract, and publication keywords. The study selection process proceeded through three levels of screening. At level 1, studies were excluded if they were reviews, letters, case reports, editorials, or comments, or if they were in languages other than English. Subsequently, another author (L.B.) assessed the selected studies for relevance, inclusion, and methodological quality. Moving to level 2, abstracts of the retained studies were reviewed for relevance, with abstracts progressing to the next screening level if they reported the clinical outcomes of series involving cholangiocarcinoma patients treated with radioembolization. For level 3 screening, full texts were obtained for relevant articles and for any citations where a decision could not be made based on the abstract alone. The quality assessment of each selected study was conducted by two investigators (MA.C. and E.D.). Any discrepancies in inclusion were resolved through discussions among the reviewers, with the participation of an additional investigator (C.M.).
